# Weighted network measures reveal differences between dementia types: An EEG study

**DOI:** 10.1002/hbm.24896

**Published:** 2019-12-09

**Authors:** Ramtin Mehraram, Marcus Kaiser, Ruth Cromarty, Sara Graziadio, John T. O'Brien, Alison Killen, John‐Paul Taylor, Luis R. Peraza

**Affiliations:** ^1^ Institute of Neuroscience Newcastle University, Campus for Ageing and Vitality Newcastle upon Tyne UK; ^2^ NIHR Newcastle Biomedical Research Centre Campus for Ageing and Vitality Newcastle upon Tyne UK; ^3^ Interdisciplinary Computing and Complex BioSystems (ICOS) research group, School of Computing Newcastle University Newcastle upon Tyne UK; ^4^ Department of Functional Neurosurgery, Ruijin Hospital Shanghai Jiao Tong University School of Medicine Shanghai China; ^5^ NIHR Newcastle in vitro Diagnostics Co‐operative Newcastle‐Upon‐Tyne Hospitals NHS Foundation Trust Newcastle upon Tyne UK; ^6^ Department of Psychiatry University of Cambridge School of Medicine Cambridge UK; ^7^ IXICO Plc London UK

**Keywords:** Alzheimer's disease, biomarker, brain connectivity, graph theory, Lewy body, Parkinson's disease, proportional thresholding

## Abstract

The diagnosis of dementia with Lewy bodies (DLB) versus Alzheimer's disease (AD) can be difficult especially early in the disease process. However, one inexpensive and non‐invasive biomarker which could help is electroencephalography (EEG). Previous studies have shown that the brain network architecture assessed by EEG is altered in AD patients compared with age‐matched healthy control people (HC). However, similar studies in Lewy body diseases, that is, DLB and Parkinson's disease dementia (PDD) are still lacking. In this work, we (a) compared brain network connectivity patterns across conditions, AD, DLB and PDD, in order to infer EEG network biomarkers that differentiate between these conditions, and (b) tested whether opting for weighted matrices led to more reliable results by better preserving the topology of the network. Our results indicate that dementia groups present with reduced connectivity in the EEG α band, whereas DLB shows weaker posterior–anterior patterns within the β‐band and greater network segregation within the θ‐band compared with AD. Weighted network measures were more consistent across global thresholding levels, and the network properties reflected reduction in connectivity strength in the dementia groups. In conclusion, β‐ and θ‐band network measures may be suitable as biomarkers for discriminating DLB from AD, whereas the α‐band network is similarly affected in DLB and PDD compared with HC. These variations may reflect the impairment of attentional networks in Parkinsonian diseases such as DLB and PDD.

## 1 INTRODUCTION

Dementia spans a range of cognitive disorders affecting almost 50 million people worldwide (American Psychiatric Association, 2013; Geser, Wenning, Poewe, & McKeith, 2005). The most common type of dementia in older adults is Alzheimer's disease (AD) dementia, with 50–70% of clinically diagnosed cases (I. McKeith et al., 2007), followed by dementia with Lewy bodies (DLB) which accounts for 4–8% of the cases (I. McKeith et al., 2007) and Parkinson's disease dementia (PDD), which develops in ≈80% of people with Parkinson's disease longitudinally (Hely, Reid, Adena, Halliday, & Morris, 2008). The main symptom of AD consists of episodic memory loss which occurs gradually, approximately within a 6‐month time frame prior seeing a clinician (Dubois et al., 2014; Grober & Buschke, 1987). Similarly to AD, cognitive impairment develops at early stages in DLB, thus misdiagnoses are a common issue (Palmqvist, Hansson, Minthon, & Londos, 2009). Detecting the early core clinical features of DLB, which include cognitive fluctuations, visual hallucinations and REM sleep behaviour disorder (I. G. McKeith et al., 2017), may increase the accuracy of the clinical diagnosis. Moreover, supportive biological indexes, also known as biomarkers, may provide additional information (I. G. McKeith et al., 2017). As stated in the “1‐year rule” (McKeith et al., 2005), 1 year after the onset of cognitive impairment, DLB patients also develop parkinsonian symptoms such as bradykinesia, tremor and rigidity (Gaig & Tolosa, 2009; Hornykiewicz & Kish, 1987); a symptomatic spectrum similar to PDD. The common aetiology of DLB and PDD is the progressive accumulation of alpha‐synuclein protein bodies across the brain, known as Lewy bodies. The overlapping causes and symptoms often lead scientists to consider these two diseases as a sole group when aiming to assess effective biomarkers for the diagnosis of dementia (Lippa et al., 2007). However, due to enhanced cognitive dysfunction preceding the motor symptoms in DLB pathology, as well as a greater accumulation of amyloid in DLB (Edison et al., 2008), physiological differences and biomarkers to differentiate DLB and PDD remain a research question and might provide further insight on the development of the two subtypes (Stylianou et al., 2018).

Electroencephalography (EEG) is emerging as a convenient technique in dementia research. It is advantageous in terms of cost (Lee & Tan, 2006), the absence of side effects and has superior temporal resolution. Previous studies using eyes‐closed resting state experimental protocol concur with the slowing of the α (alpha, 8–14 Hz) activity towards lower frequencies in DLB and PDD when compared with HC and AD. This characteristic emerges mostly in the occipital lobe (Andersson, Hansson, Minthon, Rosen, & Londos, 2008; Bonanni et al., 2015; Briel et al., 1999; Jackson & Snyder, 2008; Kai, Asai, Sakuma, Koeda, & Nakashima, 2005; Peraza et al., 2018; Stylianou et al., 2018). In particular, previous studies focused on the shifting of the dominant frequency (DF) towards slower frequencies. These studies showed that the frequency with the most prominent peak in the power spectrum moves towards a lower range of frequencies in patients when compared with healthy controls (Bonanni et al., 2008; Peraza et al., 2018; Stylianou et al., 2018). DLB related changes were also found by EEG network connectivity studies. For instance, when comparing AD and healthy participants (HC), parietal–frontal connectivity patterns, which are known to be involved in attentional processes (Corbetta & Shulman, 2002), were affected (M. Dauwan et al., 2018; Lemstra et al., 2014) in DLB participants. In a similar study based on minimum spanning tree (MST), reduced hubness, that is, lower node degree and betweeness centrality, within the α frequency band was reported by van Dellen et al. (2015) when comparing DLB with AD. The MST is obtained by preserving the minimum number of strongest edges while connecting all nodes without cycling paths. Here the authors associated the reduced hubness with a more severe cognitive impairment in DLB (van Dellen et al., 2015). In a more recent study, Babiloni and colleagues reported reduced interhemispheric connectivity patterns in dementia patients (Babiloni et al., 2018), with weaker connections in AD compared with DLB over posterior and temporal regions within the α range; this intra‐hemispheric connection showed no differences between DLB and PDD. According to the authors, this aspect is associated with the fact that the pathology is similar in both LBDs, that is, DLB and PDD. A recent work in EEG based on MST reported the α band to be discriminative between HC and dementia, whereas significant differences in the PLI strength between AD and DLB were found in the β (beta; 15–30 Hz) band (Peraza et al., 2018). Hence, the authors suggested that the β network might potentially be an EEG biomarker of DLB against AD. Connectivity was measured with phase lag index (PLI; Stam, Nolte, & Daffertshofer, 2007), a metric that is insensitive to scalp's volume conduction.

To date, no EEG studies based on proportional thresholding have been performed in order to assess network property changes related to dementia conditions including LBDs. A crucial aspect in functional network studies is how the connectivity threshold is defined in order to obtain a graph from a connectivity matrix, where the non‐relevant edges are pruned off and the edges or connections whose weights are above the threshold are preserved. At this point, a researcher may choose to binarise the matrix, that is, set to 1 all the surviving edges, or to preserve their corresponding weights. Several previous studies have dealt with the issue of network thresholding (Garrison, Scheinost, Finn, Shen, & Constable, 2015; Jalili, 2016; Langer, Pedroni, & Jancke, 2013; van Wijk, Stam, & Daffertshofer, 2010), providing rationales for each of the proposed methods. However, the choice of using weighted or binary matrices to estimate network measures has mostly been arbitrary to date. In a previous EEG network study on schizophrenia, it was shown that preserving the weights while applying network thresholding, produced more prominent differences between conditions by the network properties (Rubinov et al., 2009). Nevertheless, no further quantitative investigation has been done to date in order to assess whether preserving the EEG connection weights in dementia studies might lead to a more pronounced differentiation between groups and improve consistency of the results across network densities.

In this study, we performed an exploratory investigation of differences between dementia groups in terms of EEG connectivity patterns and strength. We also performed a graph theory analysis based on proportional thresholding to assess disease related differences between groups. In addition, we hypothesised that performing graph analysis based on proportional thresholding while preserving the weights, produces consistent results by preserving additional topological information stored in the weights.

## 2 METHODS

### 2.1 Participants

The study sample comprised 18 HC (11 male, 7 female), 32 AD (22 male, 10 female), 25 DLB (20 male, 5 female) and 21 PDD (20 male, 1 female) patients. Diagnoses were performed by two experienced clinicians according to the DLB consensus criteria (I. G. McKeith et al., 2017; I. G. McKeith et al., 2005), the diagnostic criteria for PDD (Emre et al., 2007), and the National Institute on Ageing‐Alzheimer's Association criteria for AD (McKhann et al., 2011). Clinical information was collected with a battery of neuropsychological and neuropsychiatric tests, reported in Table 1: Global cognition assessment through the Mini‐mental state examination (MMSE), the Cambridge cognitive battery tests (CAMCOG), and executive and visuo‐perceptual tests such as trail making test A, animal naming and FAS verbal fluency. Additionally, the Unified Parkinson's Disease rating scale part III (UPDRS III), cognitive assessment of fluctuation (CAF) (Walker et al., 2000) scale, and the neuropsychiatric inventory test subscale for the severity and frequency of hallucinations (NPI hallucinations) were delivered to patients. Patients with a MMSE score < 12 and healthy subjects with MMSE < 26 were excluded from the sample, which resulted in excluding one PDD patient with MMSE = 8. Levodopa equivalent daily dose (LEDD) was estimated for patients on dopaminergic medication (Tomlinson et al., 2010). All participants did not have other neurological or psychiatric conditions besides dementia in patients and gave written informed consent. This study was approved by the Northumberland Tyne and Wear NHS Trust and Newcastle ethics committee.

**Table 1:** Demographic data and clinical scores

	HC (*N* = 18)	AD (*N* = 32)	DLB (25)	PDD (21)	*p*‐value
Age	76.28 ± 5.50	76.63 ± 7.72	76.16 ± 6.24	73.38 ± 5.89	df = 3, *p*‐value = .228^a^
Male/female	11/7	11/5	20/5	40/2	df = 3, *p*‐value = .055^b^
MMSE	29.17 ± 0.86	20.16 ± 4.30	22.68 ± 4.32	23.43 ± 3.49	df = 3; *p*‐value < .001^a^
CAMCOG total	96.67 ± 3.68	66.22 ± 15.87	74.84 ± 12.78	75.86 ± 10.80	df = 3; *p*‐value < .001^a^
NPI hall	0	0.03 ± 0.18	1.71 ± 1.88	2.19 ± 1.99	*p*‐value = .312^c^
CAF total	0	0.58 ± 1.39	4.13 ± 4.13	6.63 ± 4.27	*p*‐value = .045^c^
Animal naming	20.72 ± 5.54	10.66 ± 4.97	10.80 ± 3.88	11.38 ± 4.14	df = 3; *p*‐value < .001^a^
UPDRS	1.28 ± 1.49	2.77 ± 3.11	16.20 ± 7.52	24.52± 6.71	*p*‐value < .001^c^
Angle discrimination	19.65 ± 0.86	18.23 ± 3.63	15.71 ± 4.99	17.25 ± 4.02	df = 3, *p*‐value = .004^a^
FAS verbal fluency	44.89 ± 16.07	26.43 ± 16.23	18.28 ± 10.60	20.86 ± 13.66	df = 3, *p*‐value < .001^a^
Trail making test A	36.43 ± 10.25	79.16 ± 52.55	109.88 ± 68.84	167.35 ± 107.11	df = 3, *p*‐value < .001^a^
ACheI (yes/no)	0/18	29/3	22/3	17/3^d^	df = 4, *p*‐value = .537^e^
LEDD	0	0	176.88 ± 230.44	805.90 ± 392.70	df = 44, *p*‐value < .001^c^

a Kruskal–Wallis four groups.

b χ^2^ test four groups.

c Unpaired Mann–Whitney *U* test (DLB vs. PDD).

d One PDD patient was on Memantine.

e χ^2^ test three groups (AD, DLB, and PDD).

### 2.2 Experimental protocol and EEG recording

**Figure 1:**
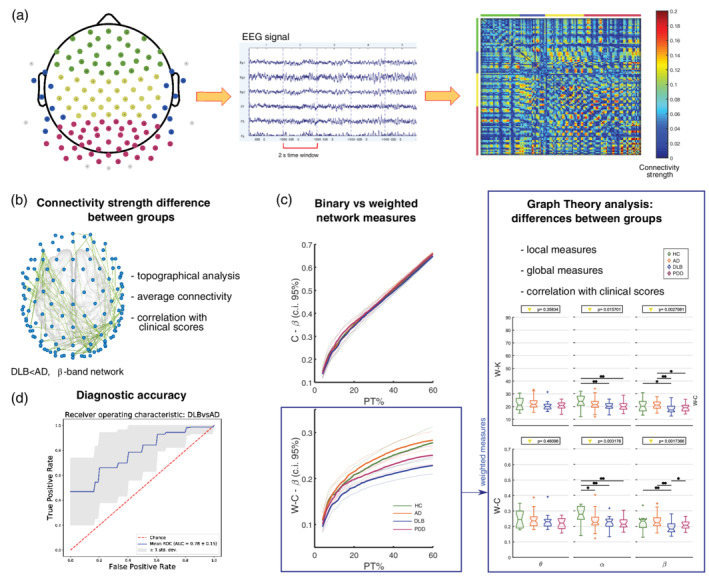
Methodological workflow. (a) From left to right: distribution of the 128 EEG electrodes in the 10‐5 system. Grey electrodes were deemed noisy, hence were excluded in all network analyses. An example of EEG recording from a healthy participant and a connectivity matrix computed on one HC subject in the β‐band network are reported. Colours span across connectivity (defined as weighted phase lag index, or WPLI, as reported in detail in section 2) values between 0 and 0.2. Coloured bars on the sides of the connectivity matrix and colour of the electrodes define scalp regions. Green: frontal region; blue: lateral region; yellow: central region; purple: posterior region. (b) Topography showing significantly weakened connections in DLB compared with AD within the β‐band network; (c) Left: binary and weighted clustering coefficient values across (β‐band) network densities, reported here as an example; right: average weighted node degree and weighted clustering coefficient, t‐tests across groups (Kruskal–Wallis value on top, *p < .05, **test survives multiple comparison correction); (d) Receiver operating characteristic curve obtained with random forest classifier, testing DLB versus AD discrimination

Participants were asked to sit in a dimly lit room and keep their eyes closed for 2.5 min. They were asked to relax while keeping awake, move as little as possible and avoid focusing on a particular thought. High density EEG with 128 sintered Ag/AgCl electrodes, 10‐5 derivation system (Figure 1a) (Robert Oostenveld & Praamstra, 2001) was recorded during the session with an EEG Waveguard cap (ANT Neuro, The Netherlands). Signals were recorded at 1,024 Hz sampling frequency and electrode impedance was kept <5 kΩ. At recording, channels were referenced to Fz and ground channel was attached to the right clavicle.

### 2.3 Pre‐processing

The EEG recordings were pre‐processed off‐line using the EEGLAB toolbox version 14 (Delorme & Makeig, 2004) on MATLAB 9.2 (The MathWorks Inc., Natick, MA, 2017). Signals were band‐pass filtered with a second‐order Butterworth filter within the range 0.5–80 Hz and a 50‐Hz notch filter was applied to remove power line noise. Time‐series were segmented in two‐second time intervals or “epochs”. Noisy or disconnected channels were removed (number of removed channels: 15 ± 13), as well as epochs showing sporadic artefacts such as muscular tension (number of removed epochs: 12 ± 10). The cleaned time series underwent independent component analysis (ICA) through the InfoMax algorithm (Bell & Sejnowski, 1995), with principal component analysis (PCA) dimension reduction to obtain a number of component equal to half the number of channels preserved in the previous step. Emerging muscular, eye and noisy components from the ICA were detected by visual inspection and rejected (number of removed components: 39 ± 10). The preserved ICA components were transformed back to time‐series domain, and the previously removed channels were interpolated by spatial spherical interpolation. All channels were referenced to spatial average.

### 2.4 Weighted phase lag index

The connectivity index chosen in this study is the weighted phase lag index (WPLI) (Vinck, Oostenveld, van Wingerden, Battaglia, & Pennartz, 2011), which is an improvement of the previous PLI statistic (Stam, Nolte, et al., 2007). The PLI measures consistency across time of the instantaneous delay between two signals through Hilbert transformations. PLI is also robust to scalp volume conduction, which is a common issue in EEG recordings (Peraza, Asghar, Green, & Halliday, 2012). The WPLI is obtained with the following equation:

(1) $$\text{WPLI}=\frac{\left|E\left\{\left|I\left\{X\right\}\right|\mathrm{sgn}\left(I\left\{X\right\}\right)\right\}\right|}{E\left\{\left|I\left\{X\right\}\right|\right\}}$$

where *X* is the cross spectrum between any couple of signals, *I(X)* is its imaginary part, *E* is the expected value and *sgn* is the sign function. In fact, this corresponds to weighting the PLI values with the imaginary part of the cross‐spectrum between the two time‐series. For the inference of brain networks, this approach reduces the influence of the almost zero‐lag connections, which may likely be due to noisy volume conducting sources (Vinck et al., 2011). The WPLI is bounded between 0 (lack of connectivity) and 1 (full synchronisation). To compute this measure, the time‐frequency representation for each EEG signal was first obtained within θ (theta, 4–7.5 Hz), α (alpha, 8–13.5 Hz) and β (beta, 14–20.5 Hz) frequency ranges (Stylianou et al., 2018) using Windowed Fourier Transform (3–10 cycles adaptive windows width, 0.5 Hz frequency step) implemented in the Fieldtrip toolbox (Oostenveld, Fries, Maris, & Schoffelen, 2011). WPLI connectivity matrices were then computed at each frequency band for all 2‐s epochs, and averaged across time. At this step, we obtained three WPLI matrices representing each frequency band for each of the participants. An example of an estimated connectivity matrix from a HC participant in the β band is shown in Figure 1a.

### 2.5 Connectivity strength

We investigated for possible bias introduced by the group's functional connectivity strength to the topology of the network. WPLI values were averaged across all edges and compared between groups. We also categorised the edges according to their length (inter‐node Euclidean distance). WPLI values were divided in four equal ranges (very short: <57 mm; short: 57–114 mm; long: 115–170 mm; very long: 171–227 mm) and differences between groups were investigated at each range.

### 2.6 Proportional thresholding

To perform graph theory analysis, we applied a proportional threshold to the connectivity matrices which preserves the edges with the highest connectivity values (weight strength). The thresholding was performed using the MATLAB function threshold_proportional.m from the Brain Connectivity Toolbox, BCT (Rubinov & Sporns, 2010). The matrices were thresholded within a range of percentage values (PT%) between 3 and 60% in steps of 1%. To be in line with the underlying structural properties of the network, a range up to 40% would already be a reasonable choice (Bohr et al., 2013; Kaiser, 2011). However, some studies also included higher densities (Giessing, Thiel, Alexander‐Bloch, Patel, & Bullmore, 2013) and in consequence, we chose a range that covered most of the choices in previous investigations. The choice of a wider range was also aimed to test the dependence of network measures on the network density (section 2.8.2).

To obtain weighted matrices, we set to 0 the values below the threshold and preserved the weights of the remaining edges. The binary matrices were obtained by setting to one all edges that survived the threshold, and to zero those edges below the threshold. Network measures were computed for each threshold level and averaged across thresholds.

### 2.7 Network measures

Local and global network measures were estimated to describe the topologies of the binary EEG networks. We also computed variants of the same measures for weighted matrices as described in the literature, in order to prove that preserving the weight strength after the thresholding step, results in a more efficient preservation of the topology of the network. Before computing the weighted measures, the matrices were normalised by dividing all the WPLI values by the maximum connectivity value within each matrix. This step resulted in having all values bounded between 0 and 1. This also aimed to remove group bias that could be introduced by group‐dependent functional connectivity strength (Onnela, Saramäki, Kertész, & Kaski, 2005). All network measures were computed with functions from the Brain Connectivity Toolbox (Rubinov & Sporns, 2010) in MATLAB, and comprised: Node degree (K), that is, average number of edges connected to a node; clustering coefficient (C), that is, average number of connections between node's neighbours; characteristic path length (L), that is, average shortest path between any pair of nodes; small‐worldness (σ), that is, the ratio between normalised clustering coefficient and characteristic path length; modularity (Q), that is, the difference between within‐ and between‐modules edges. Details on all measures are reported in Supporting Information.

### 2.8 Statistical analysis

#### 2.8.1 Connectivity strength

Statistical analyses were performed using MATLAB (Mathworks, Natick, MA; version 9). The Network Based Statistics (NBS) toolbox, version 1.2 (Zalesky, Fornito, & Bullmore, 2010), was used to estimate topographical differences of connectivity strength between groups at each frequency band. The chosen NBS threshold was set at 8 for the ANOVA test, and 3.8 for the post hoc one‐tail *t* tests, as in our data, they allowed to clearly appreciate the network topographical patterns (Zalesky et al., 2010). The family‐wise error rate (FWER) was controlled by performing a permutation test (5,000 permutations). Differences were considered significant at a *p*‐value < .05, with Bonferroni correction for the post hoc tests (12 comparisons). Networks were visualised with the BrainNet Viewer (Xia, Wang, & He, 2013). Differences in average WPLI were assessed for each frequency band with a Kruskal–Wallis test (*p* < .05) followed by post hoc two‐tailed Mann–Whitney U tests (*p* < .05) with Holm–Bonferroni correction (Holm, 1979; six comparisons). For the edge distance analysis, between‐group comparisons were performed at each frequency band for the four distance ranges. Differences across groups were assessed with Kruskal–Wallis tests (*p* < .05, Holm–Bonferroni correction for distance ranges, four tests) followed by two‐tailed Mann–Whitney U post hoc tests (*p* < .05, Holm–Bonferroni correction, six comparisons). Finally, we tested for correlations between the average WPLI and clinical scores (listed in section 2.1) for each group and frequency band by performing Spearman rank correlations; relations were considered significant at a *p*‐value < .05, uncorrected.

#### 2.8.2 Dependence of the network topology on thresholding level

To assess whether preserving the weights reduces the influence of thresholding on network topology (regardless of the group or frequency range), we performed Spearman rank correlation tests (*p* < .05) between the network measures (local measures were averaged over the whole scalp) and the 60 thresholding levels for all groups and frequency ranges together. To avoid false‐positive correlations due to the high number of observations (60 density values for each of the three frequency ranges and four participant groups), we applied a bootstrapping approach with 5,000 permutations to estimate a correlation distribution. A relation between edge density and network measure was considered significant if this was within the 0.025% of the empirical null distribution tails (|*ρ*| < .025%, i.e. double sided). We also tested for between‐group differences of the network measures at each PT% by performing a Mack–Skill test (Mack and Skillings, 1980; *p* < .05) for each frequency range. If the test resulted significant, a Kruskal–Wallis tests (*p* < .05, Holm–Bonferroni correction, 60 tests) was performed followed by two‐tailed Mann–Whitney U post hoc tests (*p* < .05, Holm–Bonferroni correction, six comparisons).

We also pursued a model fitting approach to confirm what emerges from the correlation described in the previous paragraph (Bradley, Jacob, Hermance, & Mustard, 2007; Fjell et al., 2010). To test the attenuation of the measure‐versus‐threshold dependency by preserving the weights, we fitted a power law model to the network measure‐versus‐PT% curves using the Curve Fitting toolbox (version 3.5.5) in MATLAB. We used the power law model because this resulted in lower fitting errors compared with other models such as exponential, linear or polynomial, as revealed by the sum of squares error (SSE). We then computed the first derivative, that is, the network measure dependence on the threshold level. The results obtained with this procedure are reported in section 3 for the clustering coefficient from the HC group in the β frequency range.

#### 2.8.3 Differences between groups in weighted matrices

The averaged network measures across thresholding levels were used to investigate differences between groups within each frequency band. For each measure, a Kruskal–Wallis test (*p* < .05) was performed followed by post hoc two‐tailed Mann–Whitney U tests (*p* < .05) with Holm–Bonferroni correction (six comparisons). Similar to the approach pursued in Stylianou et al.'s (2018) study, local measures were also tested regionally for differences within the frontal, temporal, central and posterior regions as shown in Figure 1a. To assess local differences between groups for each measure, we first performed a repeated measures ANOVA with region as the within subject factor and group as the between subject factor. When any interaction was found, we ran a Kruskal–Wallis test within each region (*p* < .05, Holm–Bonferroni corrected, four tests) followed by post hoc two‐tailed Mann–Whitney U tests (*p* < .05) with Holm–Bonferroni correction (six comparisons). Finally, we tested for possible rank correlations between the weighted network measures and clinical scores for each group and frequency band with Spearman tests (*p* < .05, uncorrected).

#### 2.8.4 Diagnostic accuracy

To test for the potential diagnostic utility of the most significant markers inferred in this study, we implemented a random forest classifier using the Scikit‐Learn framework in Python (version 0.20.1), and the Imbalanced‐Learn library for Python (version 0.4.3); with cross‐validation: six‐fold, ten repetitions. All the network variables, in all frequency bands, were used to train the classifier, and the mean variable importance ranking was obtained. We then computed the mean accuracy, F_1_ score, sensitivity, specificity and area under the receiver operating characteristic (AUROC) curve. Diagnostic accuracy was tested for the diagnostic scenarios that resulted with significant differences in our network analysis. Here, we only reported a six‐fold cross‐validation, but similar results are obtainable when using five‐fold or seven‐fold (Supporting Information).

## 3 RESULTS

### 3.1 Connectivity strength

**Figure 2:**
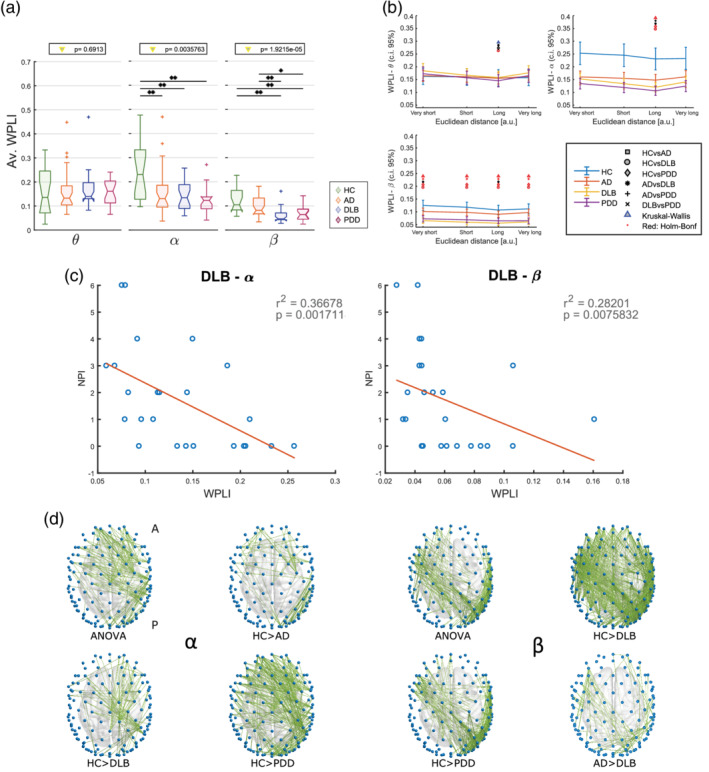
Results from the connectivity strength analysis. (a) Average WPLI for each group and frequency band; values on top indicate the result of the one‐way ANOVA (*p* < .05); *significant two‐tailed Mann–Whitney *U* test post hoc test (*p* < .05); **post hoc test survives Holm–Bonferroni correction (six comparisons). (b) Distance analysis. WPLI values are averaged by edge length ranges; very short: <57 mm; short: 57–114 mm; long: 115–170 mm; very long: 171–227 mm. Different markers were used to indicate significant results from one‐way Kruskal–Wallis (*p* < .05) and two‐tailed Mann–Whitney U test post hoc test (*p* < .05) as described in the legend on the right side. Red marker: test survives Holm–Bonferroni correction (Kruskal–Wallis: 4 ranges; post hoc: six comparisons). Error bars represent 95% confidence interval. (c) Outcome of the Spearman's test correlation. Significant correlation were found only between WPLI and NPI score in DLB at α and β frequency bands. (d) Results from the two‐tailed *t*‐tests (5,000 permutations) with the NBS (Network Based Statistics, ANOVA F‐threshold = 8, *p* < .0042; post hoc t‐threshold = 3.8, *p* < .0042) respectively in α and β range. No significant differences were found in θ band

The first part of our analysis was aimed to assess whether the pathological condition affects the connectivity strength of the network. Results from this analysis are shown in Figure 2. The average WPLI (Figure 2a) resulted weaker in the α band in all dementia groups when compared with HC, and it was reduced in LBDs compared with HC and lower in DLB compared with AD in the β‐band. The WPLI in the α‐band was significantly weaker in the dementia groups for the long connections, and for all distance ranges within the β‐band network (Figure 2b). No significant differences in WPLI between groups were found within the θ band.

In the AD group, the NBS revealed a missing right‐occipital network cluster, as well as a reduced posterior–anterior connectivity pattern and a missing frontal cluster (Figure 2d). Topographical differences in DLB consisted mostly in affected parietal–frontal connectivity. Several pathways were weakened as assessed through NBS in PDD, including bi‐lateral occipital‐frontal patterns, right‐occipital cluster and frontal connectivity. The most significant topographical differences in the β band consisted of low connectivity strength in LBDs, DLB and PDD, when compared with HC as well as a more reduced connectivity in DLB versus AD. For the β‐band network, occipital–central patterns were weakened in LBDs vs HC, as well as in the right‐temporal area. Left occipital–frontal connectivity patterns and left temporal area were weakened in DLB compared with AD (Figure 2d).

When we assessed for correlations with clinical variables, the average WPLI in DLB correlated negatively with the level of visual hallucinations (NPI‐hall score) within the α and β bands (Figure 2c). No significant correlations were found with other clinical scores.

### 3.2 Proportional thresholding

**Figure 3:**
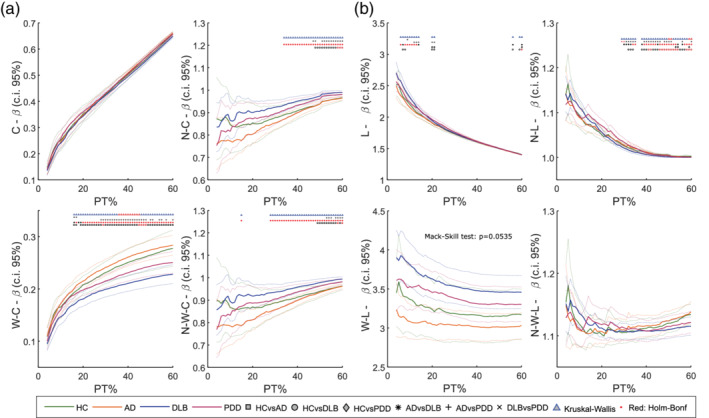
Dependence of the clustering coefficient and characteristic path length on the connectivity matrix thresholding level (PT%). Horizontal axis: PT% (range within 3–60); vertical axis: network measure. Markers on top represent results of one‐way Kruskal‐Wallis (*p* < .05) and two‐tailed Mann–Whitney *U* post hoc tests (*p* < .05) performed at each PT% as described in the legend on side. Red marker: test survives Holm–Bonferroni correction (Kruskal–Wallis: 60 tests; post hoc test: six comparisons). Dotted lines of the same colour delineate 95% confidence interval for each group. (a) From top‐left to bottom‐right: average clustering coefficient (C), average normalised clustering coefficient (N‐C), average weighted clustering coefficient (W‐C), and average normalised weighted clustering coefficient (N‐W‐C). (b) From top‐left to bottom‐right: average characteristic path length (L), normalised average characteristic path length (N‐L), weight‐based average characteristic path length (W‐L), weight‐based normalised average characteristic path length (N‐W‐L). For other weighted and binary measures, see Supporting Information

For the estimation of graph theory network measures, we first applied a proportional threshold to the WPLI matrices, preserving from 3% to 60% of the strongest connectivity values. We tested whether preserving the weights reduces the dependency of the clustering coefficient and the average characteristic path length on the thresholding level. Preservation of the weights after the thresholding step resulted in dependence attenuation of the network measures on the number of edges, as assessed with Spearman rank correlation test (*p* = 0, $\rho_{C_{b}}=0.9666,\;\rho_{C_{w}}=0.6765,\;\rho_{L_{b}}=-0.9692,\;\rho_{L_{w}}=-0.0665$). The normalised metrics were less influenced by the preservation of the weights than the not normalised ones (p = 0, $\rho_{C_{b_{\mathrm{norm}}}}=0.5134,\;\rho_{C_{w_{\mathrm{norm}}}}=0.4673,\;\rho_{L_{b_{\mathrm{norm}}}}=-0.7981,\;\rho_{L_{w_{\mathrm{norm}}}}=0.1022$). The metric‐versus‐density trends for the average characteristic path length and the average clustering coefficient in the β range, as well as the statistical tests at each thresholding level are shown in Figure 3. Statistical tests at each network density are performed only if the Mack–Skill test revealed an effect of PT% on the network measure. As revealed by the correlation tests, the curves obtained with the weighted measures show a reduced slope, that is, less dependency on the threshold axis, PT%. For the clustering coefficient (Figure 3a), significant differences between groups were found only in the weighted case (Kruskal–Wallis: *p* < .05, *t*‐test: *p* < .05 with Holm–Bonferroni correction). Particularly, the weighted clustering coefficient was significantly reduced in DLB when compared with the AD group at PT% > 15. The normalised clustering coefficient showed similar results for both the binary and weighted case, with differences between AD and DLB at PT% > 33 in the binary case and PT% > 27 in the weighted measure case. In addition, the characteristic path length dependence on the network density was strongly reduced in the weighted case (Figure 3b). Statistical tests in the weight‐based measure between groups were not dependent on the PT%, as revealed by the Mack–Skill test (*p* = .2789). In the binary case, the DLB group showed a higher W‐L compared with AD for PT% < 15. The binary normalised measure revealed differences between AD and DLB as well as between HC and DLB groups for PT% > 28. No differences between groups were found for the normalised binary L. In line with previous findings, this shows that the binarisation of the connectivity matrices may lead to loss of information related to network topology (Rubinov et al., 2009). Correlation curves for the remaining network measures are shown in Figure S1.

To obtain a further insight of the association between the weight preservation and the reduced dependence on the network density, we modelled the network‐versus‐threshold curves as first order power law equations. For this demonstration, we considered the clustering coefficient, *C*, in the HC group in the β band, but similar results are obtained in the other scenarios (see Supporting Information). For both binary and weighted measures of clustering coefficient, we modelled their edge‐density‐versus‐measure behaviour as *C*_b_ = *ft*^*g*^ + *h* and *C*_w_ = *mt*^*n*^ + *q*, with *t =* thresholding level, and *b* and *w* standing for binary and weighted measures. The model fitting resulted in the following coefficients (with the 95% confidence interval shown in brackets): *f* = 0.8065 [0.7941; 0.8189]; *g* = 0.6718 [0.6269; 0.7161]; *h* = 0.072 [0.05228; 0.09172]; *m* = 0.9912 [0.6306; 1.352]; *n* = 0.06905 [0.04059; 0.09751]; *q* = −0.6802 [−1.043; −0.3175]. The goodness of fit is described by the sum of squares error (SSE): SSE_b_ = 0.003371, SSE_w_ = 0.0002147.

By computing the first derivative of the fitting equations with respect to the thresholding level *t*, we get the dynamic of the curves, that is, the slope with respect to the thresholding level. A derivative closer to zero describes a steady behaviour. For the binary and weighted clustering coefficient we obtained: *dC*_b_/*dt* = *fgt*^*g* − 1^ and *dC*_w_/*dt* = *mnt*^*n* − 1^. We searched the values of *t* at which the weighted measures showed lower dependence on PT% compared with the binary measures. In other words, we searched a *t* at which

(2) $$\frac{dC_{w}}{dC_{b}}<1;\;0<t\leq 1.$$

By computing the ratio in (2) and replacing the corresponding coefficients, we found that the condition in Equation (2) is true when 0.0322 < *t* ≤ 1. Hence, for the clustering coefficient in healthy controls the condition expressed in (2) is true for almost all network density values.

For the remaining of this study, results will be shown for the weighted matrices only, as we now proved how these lead to more stable results than the binary ones. However, same statistics for the binary metrics are reported in Figure S4. Network measures computed using non‐thresholded weighted matrices are also reported in Figures S5 and S6.

### 3.3 Network properties

**Figure 4:**
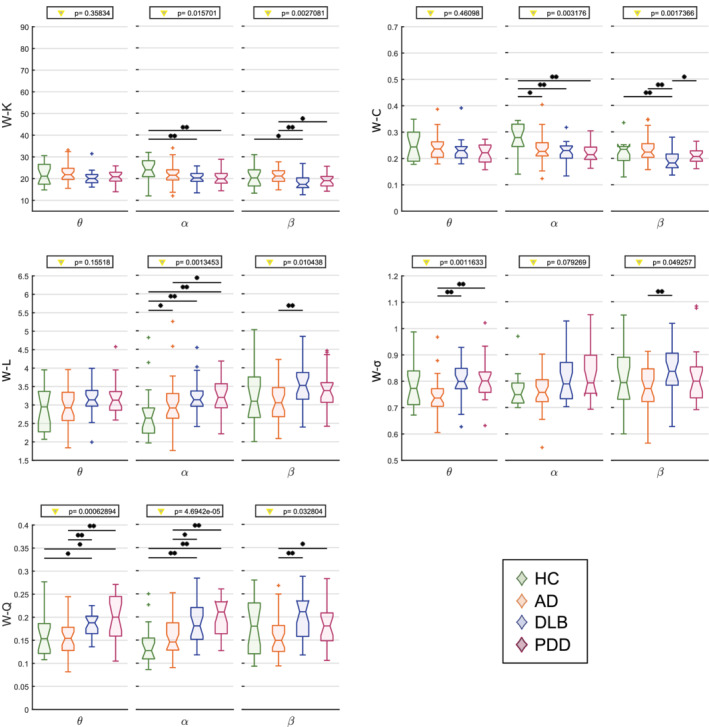
Results from the graph theory analysis on the average weight‐based network measures. Vertical axis: network measure. W: weighted. K: node degree; C: clustering coefficient; L: characteristic path length; σ: small‐worldness; Q: modularity. Horizontal axis: frequency band of interest (θ: 4–7.5 Hz, α: 8–13.5 Hz, β: 14–20.5 Hz). Values on top indicate the result from the one‐way Kruskal–Wallis test (*p* < .05); *significant two‐tailed Mann–Whitney U test post hoc test (*p* < .05); **post hoc test survives Holm–Bonferroni correction (six comparisons)

We hypothesised that the architecture of the EEG network at rest is affected due to the different subtypes of dementia. Results from the network measure comparisons between groups at each frequency range are shown in Figure 4. Differences between groups within the θ band were found only for the small‐worldness and modularity indices. The LBD groups (DLB and PDD) showed an increased network segregation, when compared with the AD group. Furthermore, this network segregation strongly correlated with cognitive scores (MMSE and CAF) and the NPI‐hall score in the same frequency band in PDD, although it did not in DLB. Significant differences were found in all measures (and a trend for the small‐worldness index) between LBDs and HC within the α range. The nodal measures were lower in patient groups, reflecting the differences in the connectivity strength reported above. Also network integration was reduced in all dementia groups, as reflected by the higher characteristic path length and modularity. The average clustering coefficient and the average characteristic path length respectively in DLB and PDD groups correlated with the Animal naming test, whereas the node degree and the average characteristic path length in DLB were associated with the verbal fluency (FAS) test score. The strongest difference was found within the β network and comprised a greater general alteration of the network in the DLB group when compared with the AD group for all the network measures. In this regard, DLB patients showed weaker connectivity and more segregated networks compared with AD ones, with subtle differences with PDD and HC participants. Values and plots for correlations with clinical scores are shown in Table 2 and Figure S7.

**Table 2:** Correlations between network measures and clinical scores as assessed by Spearman test (*p* < .05, uncorrected)

		HC			AD			DLB			PDD		
		*θ*	*α*	*β*	*θ*	*α*	*β*	*θ*	*α*	*β*	*θ*	*α*	*β*
WPLI	*NPI*	–	–	–	–	–	–	−0.29 (0.165)	**−0.61 (0.002)**	**−0.53 (0.008)**	0.02 (0.936)	−0.21 (0.362)	−0.23 (0.325)
W‐K	*FAS*	−0.01 (0.974)	−0.35 (0.154)	−0.44 (0.070)	0.07 (0.705)	0.03 (0.886)	−0.13 (0.493)	−0.26 (0.215)	**−0.46 (0.020)**	−0.36 (0.081)	−0.10 (0.666)	−0.19 (0.400)	−0.26 (0.250)
W‐C	*Animals*	−0.46 (0.056)	−0.18 (0.465)	0.14 (0.578)	−0.06 (0.740)	0.32 (0.073)	−0.18 (0.315)	−0.10 (0.639)	**−0.44 (0.028)**	−0.13 (0.524)	−0.12 (0.617)	−0.43 (0.050)	−0.11 (0.627)
W‐L	*FAS*	0.04 (0.871)	0.34 (0.173)	0.42 (0.086)	−0.06 (0.734)	−0.00 (0.981)	0.17 (0.384)	0.30 (0.147)	**0.44 (0.030)**	0.36 (0.073)	0.06 (0.781)	0.16 (0.499)	0.28 (0.215)
	*Animals*	0.38 (0.115)	0.09 (0.733)	−0.28 (0.270)	0.06 (0.742)	−0.16 (0.388)	0.32 (0.075)	0.12 (0.553)	0.39 (0.057)	0.18 (0.380)	0.24 (0.300)	**0.45 (0.041)**	0.15 (0.504)
W‐σ	*MMSE*	−0.36 (0.142)	−0.09 (0.724)	−0.44 (0.070)	0.21 (0.254)	−0.25 (0.160)	0.12 (0.508)	−0.06 (0.782)	0.12 (0.571)	0.14 (0.501)	**−0.62 (0.003)**	−0.37 (0.101)	−0.01 (0.968)
	*CAF*	–	–	–	–	–	–	0.09 (0.691)	−0.31 (0.138)	−0.09 (0.684)	**0.56 (0.014)**	0.43 (0.064)	0.06 (0.818)
	*NPI*	–	–	–	–	–	–	0.22 (0.292)	0.17 (0.428)	0.37 (0.077)	**0.49 (0.025)**	0.32 (0.151)	−0.16 (0.499)
	*UPDRS*	0.02 (0.949)	−0.18 (0.478)	−0.17 (0.497)	−0.20 (0.290)	0.11 (0.547)	0.04 (0.844)	**0.40 (0.049)**	−0.04 (0.842)	−0.17 (0.421)	0.31 (0.165)	0.38 (0.093)	−0.16 (0.476)
W‐Q	*MMSE*	−0.17 (0.491)	−0.04 (0.869)	−0.31 (0.217)	0.19 (0.287)	−0.26 (0.157)	0.06 (0.746)	−0.17 (0.425)	0.27 (0.196)	0.16 (0.440)	**−0.74 (0.0001)**	−0.37 (0.102)	−0.07 (0.777)
	*CAF*	–	–	–	–	–	–	0.21 (0.328)	−0.19 (0.371)	0.06 (0.770)	**0.69 (0.001)**	0.40 (0.092)	−0.02 (0.951)
	*FAS*	0.003 (0.99)	**0.52 (0.026)**	0.33 (0.177)	0.34 (0.063)	0.02 (0.932)	0.13 (0.495)	−0.22 (0.286)	0.31 (0.137)	0.03 (0.891)	−0.09 (0.711)	−0.03 (0.913)	0.01 (0.982)

*Notes*: *ρ* and *p* values are shown whether any correlation for that network measure and clinical variable was found for any diagnosis group and frequency band. Significant correlations are highlighted in bold.

**Figure 5:**
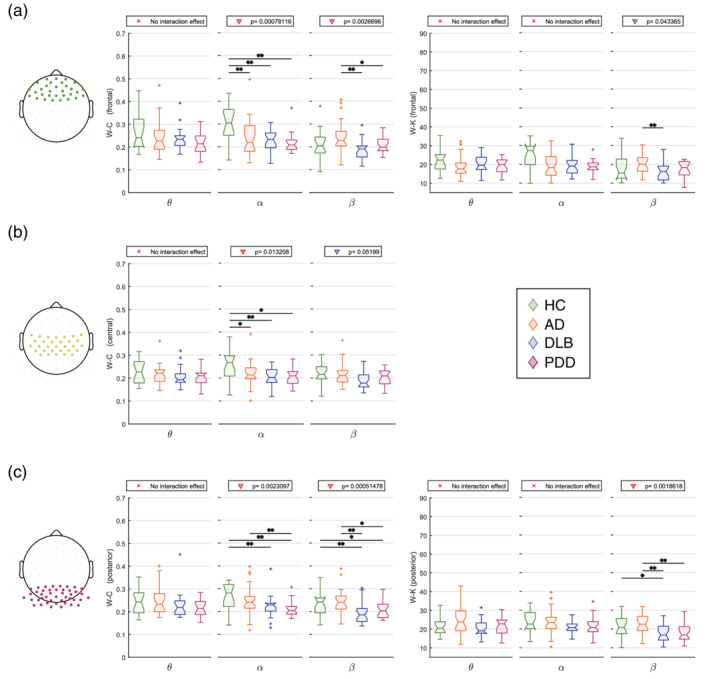
Results from the local graph theory analysis through average local weight‐based network measures. *Y*‐axis: local network measure; x‐axis: frequency band of interest (θ: 4–7.5 Hz, α: 8–13.5 Hz, β: 14–20.5 Hz). If any interaction was found in the repeated measures ANOVA (within subjects: areas; between subjects: group), the result of the one‐way Kruskal–Wallis test (*p* < .05) is indicated on top of each plot. Red triangle: Kruskal–Wallis test survives Holm–Bonferroni correction (four areas); *significant two‐tailed Mann–Whitney U test post hoc test (*p* < .05); **post hoc test survives Holm–Bonferroni correction (six comparisons). (a) Frontal area. (b) central area. (c) posterior area. No significant differences between groups were found in the lateral area and for the node degree in the central area

We also looked at regional differences in the local measures (average node degree and average clustering coefficient; Figure 5). The node degree did not show any local difference between groups in the α range, whereas the general alteration in the DLB group compared with AD within the β band was prominently driven by the occipital region (*p* < .01), although the frontal region also resulted significant (*p* < .05). For the node degree, significant differences in the clustering coefficient between DLB and AD groups were found within the β band in frontal and posterior areas. The α network was affected in the frontal, central and posterior areas. For both measures, the lateral areas were not affected by the disease. The local changes of the node degree and clustering coefficient reflect the connectivity patterns associated with network disruption, see the Connectivity Strength section.

### 3.4 Diagnostic accuracy

**Figure 6:**
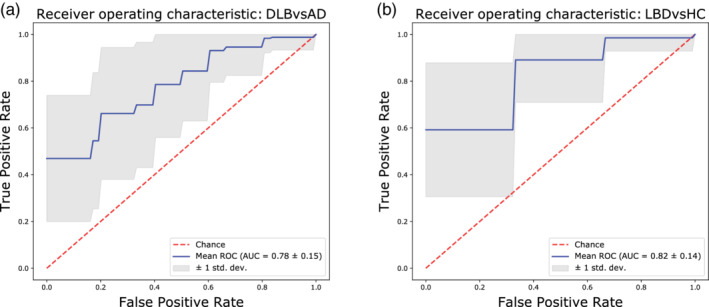
Receiver operating characteristic (ROC) curves obtained by the random forest classifier and computed for each of the defined scenarios. All (weighted) network measures were used to train the classifier. (a) DLB versus AD, mean accuracy: 66% (± 13), optimal sensitivity and specificity, respectively, of 47 and 100%; (b) LBD versus HC, mean accuracy: 76% (± 12%), optimal sensitivity and specificity respectively of 59 and 100%

For the classification analysis, we investigated two scenarios where network properties were significantly different between groups for most network parameters: DLB versus AD and LBDs versus HC. Results for other scenarios are reported in Supporting Information. All weighted network measures were used to perform a random‐forest classification, and to compute the receiver operation characteristic (ROC) curves shown in Figure 6. For each classification, a mean variable importance ranking was obtained. For the first classification (DLB vs. AD), we found a mean accuracy of 66% (± 13), mean F_1_ score of 65% (± 13%), mean positive predictive value (PPV) of 66% (± 22%), mean negative predictive value (NPV) of 71% (± 13.04%), an optimal sensitivity and specificity respectively of 47 and 100%, and area under the curve (AUROC) of 78% (± 15%). The four most important variables as ranked by the classifier were the WPLI in the β band, the modularity index in the θ band, the node degree in the β band, and the small‐worldness index in the θ band. For the second scenario, LBDs versus HC, the classifier gave a mean accuracy and F_1_ score of 76% (± 12%), a mean PPV of 88% (± 10%), mean NPV of 59% (± 21%), an optimal sensitivity and specificity respectively of 59 and 100% and AUROC of 82% (± 14%). The four most important variables, as ranked by the classifier, were the WPLI in the β band, the modularity, characteristic path length and clustering coefficient in the α band.

## 4 DISCUSSION

In this study, we hypothesised that the different dementia subtypes are associated with different alterations in their EEG network architecture. Connectivity strength resulted weakened in the dementia groups compared with the HCs in the α band, and this was significantly altered in DLB compared with AD in the β band. The difference in terms of connectivity strength between groups translated into a bias in the network architecture measurements, which we took into account. We showed that weighted measures produce consistent results in a graph theory study, where an altered β‐band network in DLB compared with AD emerged as the most significant result. Moreover, the brain network in DLB and PDD (both LBDs) were more affected compared with the HCs, and showed a higher segregation compared with the AD group. The classification between DLB and AD, performed with the random forest approach, was driven by connectivity strength and node degree in the β band as well as by network segregation in the θ band. For the LBDs, these differentiated from the HC group by their connectivity strength in the β band and the graph properties in the α band.

Patients in our study were on medication. This might have partially restored the EEG activity towards healthier values (Agnoli, Martucci, Manna, Conti, & Fioravanti, 1983; Balkan et al., 2003). Nevertheless, we found significant alterations across patient groups and our findings resonated with results from previous studies (Peraza et al., 2018; Stam et al., 2009).

### 4.1 Connectivity strength

The first step in this study was to assess whether participant groups showed differences in terms of overall functional connectivity strength driven by the pathological condition, and correct for this before the estimation of network measures. This was necessary as it has been previously shown that functional connectivity strength may introduce a bias in the network measures (van den Heuvel et al., 2017).

#### 4.1.1 Average connectivity is reduced in dementia

Statistical analysis for the average connectivity across groups revealed that the WPLI within the α frequency band was significantly reduced in patient groups compared with HCs. Our analysis also showed that the overall connectivity is weakened in the β band for all groups, but significantly reduced in LBDs compared with HCs. This also revealed that the β band could be a potential biomarker to differentiate between the AD and DLB groups. This latter finding is in line with previous M/EEG connectivity studies (Dauwan et al., 2016; Engels et al., 2015; Peraza et al., 2018; Stam et al., 2009), and may be associated with a more randomised structure of the network in LBDs (Peraza et al., 2018). Interestingly, the differences between groups within the α and β band reproduced the scenario found in a previous fMRI study for the distance analysis, where they found a decreasing trend of connectivity strength in longer connections (Peraza, Taylor, & Kaiser, 2015). In addition, the WPLI values correlated negatively with the visual hallucination score assessed by NPI‐hall in DLB for both α and β ranges. This latter finding supports a previous study which pursued a modelling approach to associate visual hallucinations with impairment of the attentional networks in LBDs (Shine, Halliday, Naismith, & Lewis, 2011) and it is in line with the role of EEG α and β frequency activity in attentional mechanisms, and the α band activity in visual processes (Anderson & Ding, 2011; Bauer, Kennett, & Driver, 2012; Lopes da Silva, 2013).

#### 4.1.2 Topographical connectivity patterns are altered in dementia

The NBS analysis revealed that the differences in connectivity strength were driven by the disruption of posterior–anterior networks in AD and DLB when compared with HCs, in agreement with previous findings (Dauwan et al., 2016; Lemstra et al., 2014). This matches as well with the outcome from the distance analysis, which revealed that the most prominent differences are observable in both α and β frequency ranges for the longest edges (Figure 2b). We hypothesise that the weakening of the posterior–anterior connections is associated with impairment of the attentional networks, which are known to be affected in AD and DLB (Corbetta & Shulman, 2002; Cromarty et al., 2018). We believe that the disruption of the occipital brain network may play a role in the alteration of the information flow towards the frontal area in DLB (Bonanni et al., 2008; Briel et al., 1999; Peraza et al., 2014).

Our results partially contrast with a recent EEG connectivity study where differences between dementia groups were found in the α band, and no differences were found in the β band (van Dellen et al., 2015). This apparent contrast may be due to methodological differences in the analysis. In particular, the use of PLI as connectivity measure might omit significant differences between groups in scenarios when the overall connectivity is low, such as we found in the β band.

### 4.2 Weighted measures preserve topological information

Our results showed that preserving the weights of the connectivity matrix prevents the loss of topological network information. As reported previously in a similar research work in schizophrenia (Rubinov et al., 2009), weighted measures revealed more prominent differences between patient groups than when compared with binary ones. Contrary to what has been stated in previous studies (Li et al., 2009; Ponten, Douw, Bartolomei, Reijneveld, & Stam, 2009; van Wijk et al., 2010), we found that the outcome of the analysis is influenced by the weights. However, in Ponten et al. (2009) the authors only considered the network measures normalised by random surrogates when comparing binary and weighted matrices. We found that network normalisation reduces the dependence of the network on the weights (Figure 3). However, the normalisation may introduce a bias that accentuates the size effect on the measures (van Wijk et al., 2010).

### 4.3 Weighted measures are less dependent on network density

The dependence of the network measures on the edge density is well a reported issue (Langer et al., 2013; van Wijk et al., 2010). By showing that the preservation of the weights makes the measures more consistent across network densities, we provide a rationale for the use of thresholded weighted matrices rather than binary ones in graph theory studies. One may consider instead not to threshold the matrix and work with the entire weighted matrix. In this latter case, the interpretation of the connectivity measures would not be the same. For instance, the node degree would become an indication of the total involvement of the node in the network, rather than the number of connected nodes (Opsahl, Agneessens, & Skvoretz, 2010). Hence, we believe that thresholding the network while preserving the weights would be a reasonable compromise. Another strategy for removing the dependence on network density is the definition of graphs based on MST. As mentioned earlier, this approach leads to fully connected weighted graphs (Stam et al., 2014), resulting in full sized networks. In fact, Peraza et al. (2018) performed a study using the MST on the same cohort of participants. However, although the differences in connectivity strength are comparable with our findings, the MST approach does not provide local alterations within the network architecture, which we found significant in patient groups.

Our approach presents with some limitations, as these results should be considered limited to the context of EEG connectivity analysis. Further investigations will be required to reproduce our results with other functional connectivity approaches. Moreover, the choice of the connectivity measure may have influenced the outcome of this analysis. WPLI attenuates spurious edges by de‐weighting the connections between signals with small phase difference (Vinck et al., 2011). This leads to a reduced influence of a larger number of weak edges when these are added to the matrix as the edge density is increased.

### 4.4 The brain functional network is segregated in dementia

The brain network in the dementia groups was more segregated and less integrated. The reduced integration is reflected by the increased characteristic path length, in line with a previous study in EEG (Stam, Jones, Nolte, Breakspear, & Scheltens, 2007). A longer path length may be associated with a reduced interaction between cortical areas (Sporns & Zwi, 2004), however, this contrasts with another investigation where the path length in the AD group was shorter than the HC group (de Haan et al., 2009). The strategy pursued by thresholding the network as well as the choice of the connectivity measure may play a key role in the interpretation of the obtained results. In de Haan and colleagues’ work, three arbitrary threshold values were chosen to compute the network properties, while in our study, a wider range of thresholding values was considered, which added more information related to the network architecture. In addition, they opted for a binarised and normalised definition of this measure. In fact, de Haan and colleagues claimed that the reduction of the normalised characteristic path length reflects loss of hubness and more randomised network topology in AD, which agreed with previous studies (de Haan, Mott, van Straaten, Scheltens, & Stam, 2012; Stam, 2014) and with our results (section 4.5).

The network segregation in the LBD groups emerged particularly within the θ band for small‐worldness and modularity, in line with a previous study on fMRI performed on the same participant cohort (Peraza et al., 2015). This phenomenon is associated with the presence of a larger number of short‐range connections altogether with a weakening of the longest connections in dementia groups, as we found in the distance analysis (Figure 2b), and which led to a higher normalised clustering coefficient and to a higher small‐worldness. We also found that network segregation in the θ band strongly correlated with the clinical scores associated with cognitive abilities (MMSE and CAF) in PDD. Previous studies attributed to the θ‐band activity a role in memory consolidation processes, modulation of information transfer and integration across different regions (Lopes da Silva, 2013). We may then speculate that these processes might be affected in PDD, but not in DLB. Nevertheless, further analysis will be needed in order to assess why this strong correlations did not emerge in DLB, and to interpret the correlation between the graph measures and the animal naming and the FAS tests reported in section 3 (Table 2).

### 4.5 Network hubness is reduced in dementia

We also found reduced node degree and clustering coefficient in LBDs in the α band and for DLB versus AD in the β band. As also reported in a previous study, this finding may reflect a reduced hubness of the network due to the pathological condition (Engels et al., 2015), as we also found via targeted node attack (see Supporting Information), driven by posterior and frontal regions (Figure 5), which perfectly resonates with the connectivity disruption patterns reported in the connectivity strength section above. As mentioned, the EEG α and β frequency bands are known to have a major role in attentional processes (Anderson & Ding, 2011; Bauer et al., 2012; Lopes da Silva, 2013), which let us speculate that the impairment of the corresponding networks may be associated with the changes of measured connectivity metrics. However, in the α range, no local differences were found for the node degree, and the clustering coefficient was also affected in the central region in DLB.

### 4.6 LBDs versus HC groups’ classification shows high accuracy

The best discrimination using a random forest classifier was obtained between the LBDs and HC groups (AUROC = 0.82 ± 0.14). The results obtained with the classifier reflect the outcome of the graph theory analysis, as the β and α band network measures resulted most discriminative. In particular, the metric that mostly drove the classification was WPLI in the β band. As mentioned above, this may highlight the role of the randomisation of the network in LBDs associated with the pathology (Peraza et al., 2018).

### 4.7 Higher segregation and reduced hubness discriminate DLB from AD

The importance of the WPLI within the β range in discriminating Lewy body diseases emerges also in the DLB versus AD scenario (AUROC = 0.78 ± 0.15). Moreover, the higher segregation of the θ network as well as the lower node degree in the β band network for the DLB group were also crucial. These results resonate with findings from the statistical comparisons between groups discussed in the previous paragraphs, and suggest that the EEG network measures within the β and θ band may be potential biomarkers for DLB versus AD differentiation. The outcome of the classification analysis confirms that the more randomised network structure in DLB is a prominent alteration compared with AD. In fact, the increased segregation and reduced hubness in DLB suggest that this can be described as a more severe disconnection syndrome compared with AD (de Haan et al., 2009; Delbeuck, Van der Linden, & Collette, 2003).

### 4.8 The optimal working point of the classifier corresponds to maximum specificity

Surprisingly, the optimal point of the classifier, that is, the point on the ROC curves at which the difference between true and false positive values was the highest (Fluss, Faraggi, & Reiser, 2005; Perkins & Schisterman, 2005), corresponded to the maximum specificity (100%) and lowest sensitivity (47 and 59%, respectively, for the two scenarios). The choice of the optimal point is a matter of debate among researchers, and new studies are proposing alternative methods whose choice might be more clinically relevant (Rota & Antolini, 2014; Unal, 2017; Zou, Yu, Liu, Carlsson, & Cabrera, 2013). In our study, we opted for the most common strategy. However, the discrete size of our sample as well as the imbalanced distribution of subjects among groups might have affected the outcome of the classification (Brereton, 2006; Sun, Wong, & Kamel, 2009).

### 4.9 The connectivity strength is the most important discriminatory variable

The higher relevance of the connectivity strength compared with other network measures in discriminating the forms of neurological disease was also found in previous studies (Peraza et al., 2018; Xu et al., 2016). In this sense, our finding provide further evidence to the fact that it is likely that simpler measures such as connectivity weights might be accurate enough for diagnostic purposes. This strengthens the suitability of EEG as a clinical diagnostic tool. Nevertheless, graph network measures might reveal alterations associated with the severity of the disease. Future studies involving prodromal and larger cohorts will be required to explore whether the network changes reported in this study may also predict the development of the disease.

## 5 CONCLUSION

In this study, we found that the connectivity strength and node degree as well as the network segregation in the β‐band (14–20.5 Hz) and the θ‐band (4–7.5 Hz) differentiated DLB versus AD. Furthermore, the network measures in the α‐band (8–13.5 Hz) were significantly affected in LBDs compared with HCs. We also demonstrated that performing an EEG graph theory analysis while preserving the weights from the connectivity matrices after the proportional thresholding step, leads to more consistent results across network densities. Therefore, we provided a rationale for choosing this approach rather than working with binary adjacency matrices, which results in the suppression of information stored in the weights. We believe that our findings altogether with the advantageous properties of EEG as a recording system, suggest that EEG has potential to become a clinical diagnostic tool for dementia.

## Supporting information


**Appendix S1**: Supplementary material

## Data Availability

The data that support the findings of this study are available from the corresponding author, upon reasonable request.
