# Supplementary material for: Weighted network measures reveal differences between dementia types: An EEG study
Source: Hum Brain Mapp. 2019 Dec 9;41(6):1573–90. doi: 10.1002/hbm.24896 (PMC7267959; doi:10.1002/hbm.24896)
Supplement: Supplementary file 1 — Appendix S1: Supplementary material [file HBM-41-1573-s001.docx]

Supplementary material:

Weighted network measures reveal differences between dementia types: an EEG study

Authors:

Ramtin Mehraram, Marcus Kaiser, Ruth Cromarty, Sara Graziadio, John T. O’Brien, Alison Killen, John-Paul Taylor, and Luis R Peraza

Contents

1. Proportional thresholding: binary measures
2. Network measures: local and global metrics
3. Network properties: binary measures
4. Network properties: weighted non-thresholded matrices
5. Correlation between network measures and clinical scores
6. Robustness of the network
7. Diagnostic accuracy
8. Connectivity and network measures: 10-20 system
9. Proportional thresholding: binary measures

Figure S1 shows the trends of all network measures in our study (excluding C and L in the β-band network, reported in the main manuscript) with respect to the network density.

Spearman correlation test between the network measures and the thresholding level for all frequency ranges and groups together: $\rho_{K}=1 \left( p=0 \right);\rho_{K_{w}}=0.9105 \left( p=0 \right);\rho_{Q}=-0.4727 \left( p=0 \right);\rho_{Q_{w}}=-0.4254 \left( p=0 \right);\rho_{\sigma}=0.6424 \left( p=0 \right);\rho_{\sigma_{w}}=0.4754 (p=0)$.

Figure S1 - Dependence of the network measures on the connectivity matrix thresholding level (PT%). Horizontal axis: PT% (range within 3% to 60%); vertical axis: network measure. Markers on top represent results of the one-way Kruskal-Wallis (p<0.05) and two-tailed Mann-Whitney U post-hoc tests (p<0.05) performed at each PT%, as described in the figure’s legend (bottom right). Red marker: test survives Holm-Bonferroni correction (Kruskal-Wallis: 60 tests; post-hoc test: 6 comparisons). Dotted lines of the same colour delineate 95% confidence interval for each group. Weighted clustering coefficient and characteristic path length plots in the β-band are reported in the main manuscript.

For both binary and weighted characteristic path length measures (clustering coefficient is reported in the main manuscript), we modelled their edge-density vs value behaviour curves as $L_{b}=ft^{g}+h$ and $L_{w}=mt^{n}+q$, with *t=* thresholding level; *f=-5.27 [-6.206; -4.333]; g=0.09554 [0.07464; 0.1164]; h=6.431 [5.48; 7.381]; m=0.03879 [0.02946; 0.04813]; n=-0.8438 [-0.8974; -0.7902]; q=3.086 [3.063; 3.109]* (Figure S2 and Figure S3)*.* The goodness of fit described by the sum of squares error (SSE) was SSE_b_=0.01649, SSE_w_=0.06544. Numbers in square brackets represent the 95% confidence interval.

By computing the first derivative of the equations with respect to the thresholding level *t*, we obtained: $dL_{b}/dt=fgt^{g-1}$ and $dL_{w}/dt=mnt^{n-1}$. As in the main manuscript, we searched the values of *t* at which the weighted measure showed lower dependence on PT% compared with the binary measure. In other words, we search a *t* at which

$\frac{dL_{w}}{dL_{b}}<1; 0<t\leq1$. (1)

By computing the ratio in (1) and replacing the corresponding coefficients, we found that the condition in equation (1) is true when $0.0544<t\leq1$. Hence the condition expressed in (1) is true for almost all network density values for the characteristic path length.

Figure S2 - Power law fitting curve for the binary characteristic path length L. y-axis: L; x-axis: network density. Black dots: average experimental L across subjects. Blue line: fitting curve.

Figure S3 - Power law fitting curve for the weight-based characteristic path length L. y-axis: W-L; x-axis: network density. Black dots: average experimental L across subjects. Blue line: fitting curve.

1. Network measures: local and global metrics

2.1 Local network measures

Studied local network measures, i.e. nodal measures, comprised the node degree K and the local clustering coefficient C (Watts and Strogatz, 1998). The degree of a node indicates the number of other nodes directly connected to it (neighbours), i.e. the number of edges connected to the node. The weighted variant of the node degree (hereafter weighted node degree) W-K is computed as an integration of the connectivity strength of all edges connected to the node (Opsahl, et al., 2010). In general, for the same node the weighted degree is lower than the degree from the binary matrices. As result, a highly connected region (in terms of strength and number of connections) is identified by a high average node degree. The clustering coefficient of a node is computed as a ratio between the number of actual connections and the number of all possible connections between neighbours (Kaiser, 2011). The weighted clustering coefficient, W-C, is computed by weighting the clustering coefficient by the sum of the intensities of the subnetworks formed by the inter-neighbour edges of each node (Onnela, et al., 2005). Contrary to its binary counterpart, the weighted clustering coefficient is sensitive to weakly connected neighbours (Onnela, et al., 2005). This is the case for weighted connectivity matrices where the edge weights span between 0 (not connected) and 1 (fully connected), rather than switching between these two values as for binary matrices. Both clustering indices binary and weighted, are bounded between 0 (neighbours not connected to each other) and 1 (all the neighbours are fully connected to each other).

2.2 Global network measures

The global network measures included the average characteristic path length L, the small-worldness (Humphries and Gurney, 2008) σ and the modularity index Q (Newman and Girvan, 2004). The characteristic path length of any couple of nodes is the length of the shortest path between the two nodes. In the binary case, the distance is computed as the lower number of intermediate nodes tracing a path between the pair of interest. In weighted matrices, the computation of the paths is made by assuming an inverse relation between weights and connection lengths (Dijkstra, 1959; Newman, 2001). For both cases, the Dijkstra algorithm is used to compute the shortest path between every pair of nodes (Dijkstra, 1959). The small-worldness is an index of network segregation (Humphries and Gurney, 2008). We computed this measure using both the binary and weighted measures. In order to estimate the small-worldness, the clustering coefficient and the average characteristic path length need to be normalised by the same measures computed from equivalent random matrices. To obtain the normalised measures, we divided the clustering coefficient and the average characteristic path length (binary and weighted) by the average of the same measures obtained from 40 equivalent random networks that preserved the node degree distribution of the real networks (Humphries and Gurney, 2008; Sanz-Arigita, et al., 2010). We also estimated network modularity. This measure is greater in networks with dense connectivity within specific node communities, and it is computed as the difference between the fraction of edges within modules and the expected fraction of randomly distributed edges. Network modularity is bounded between -1 and 1. For a review on network measures and statistics see (Rubinov and Sporns, 2010) and (Kaiser, 2011).

1. Network properties: binary measures

_
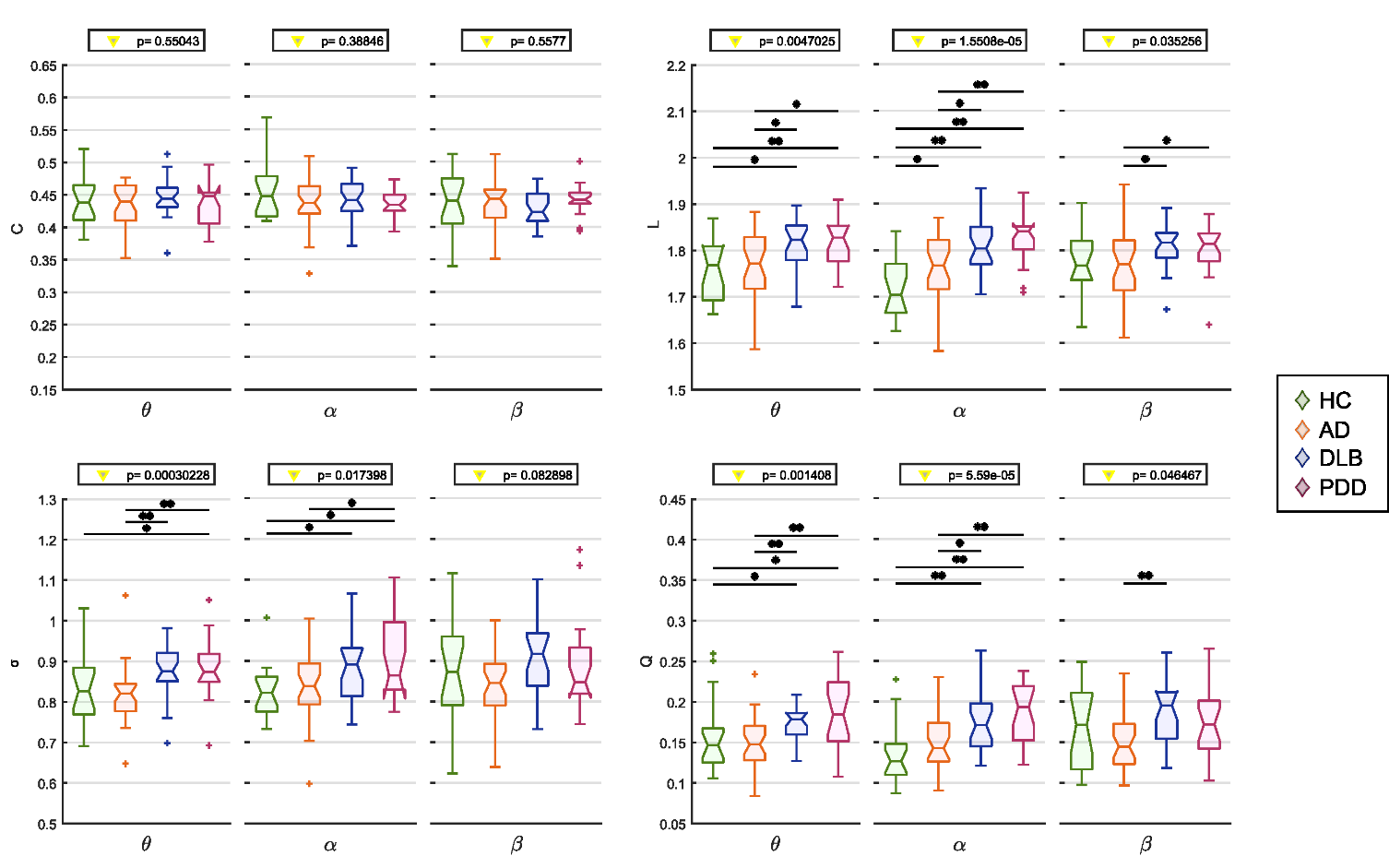
_

Figure S4 - Results of the graph theory analysis from the binary network measures. y-axis: network measure; x-axis: frequency band of interest (θ: 4-7.5 Hz, α: 8-13.5 Hz, β: 14-20.5 Hz). Values on top indicate the result of the one-way Kruskal-Wallis test (p<0.05); * : significant two-tailed Mann-Whitney U test post-hoc test (p<0.05); ** : post-hoc test survives Holm-Bonferroni correction (6 comparisons).

1. Network properties: weighted non-thresholded (complete) matrices


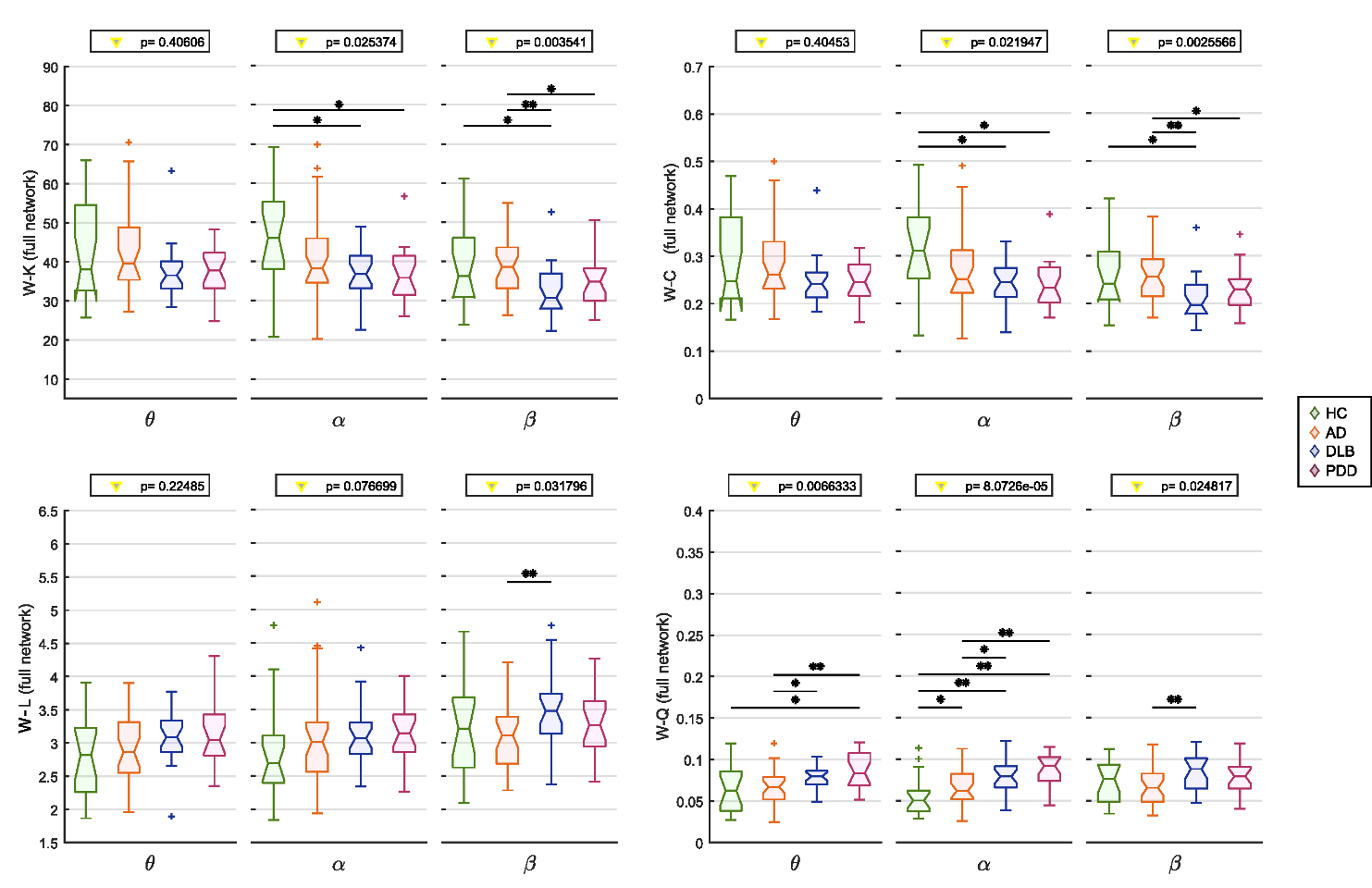


Figure S5. Results from the graph theory analysis from the weighted measures computed over non-thresholded networks. x-axis: network measure; y-axis: frequency band of interest (θ: 4-7.5 Hz, α: 8-13.5 Hz, β: 14-20.5 Hz). Values on top indicate the result of the one-way Kruskal-Wallis test (p<0.05); * : significant two-tailed Mann-Whitney U test post-hoc test (p<0.05); ** : post-hoc test survives Holm-Bonferroni correction (6 comparisons).


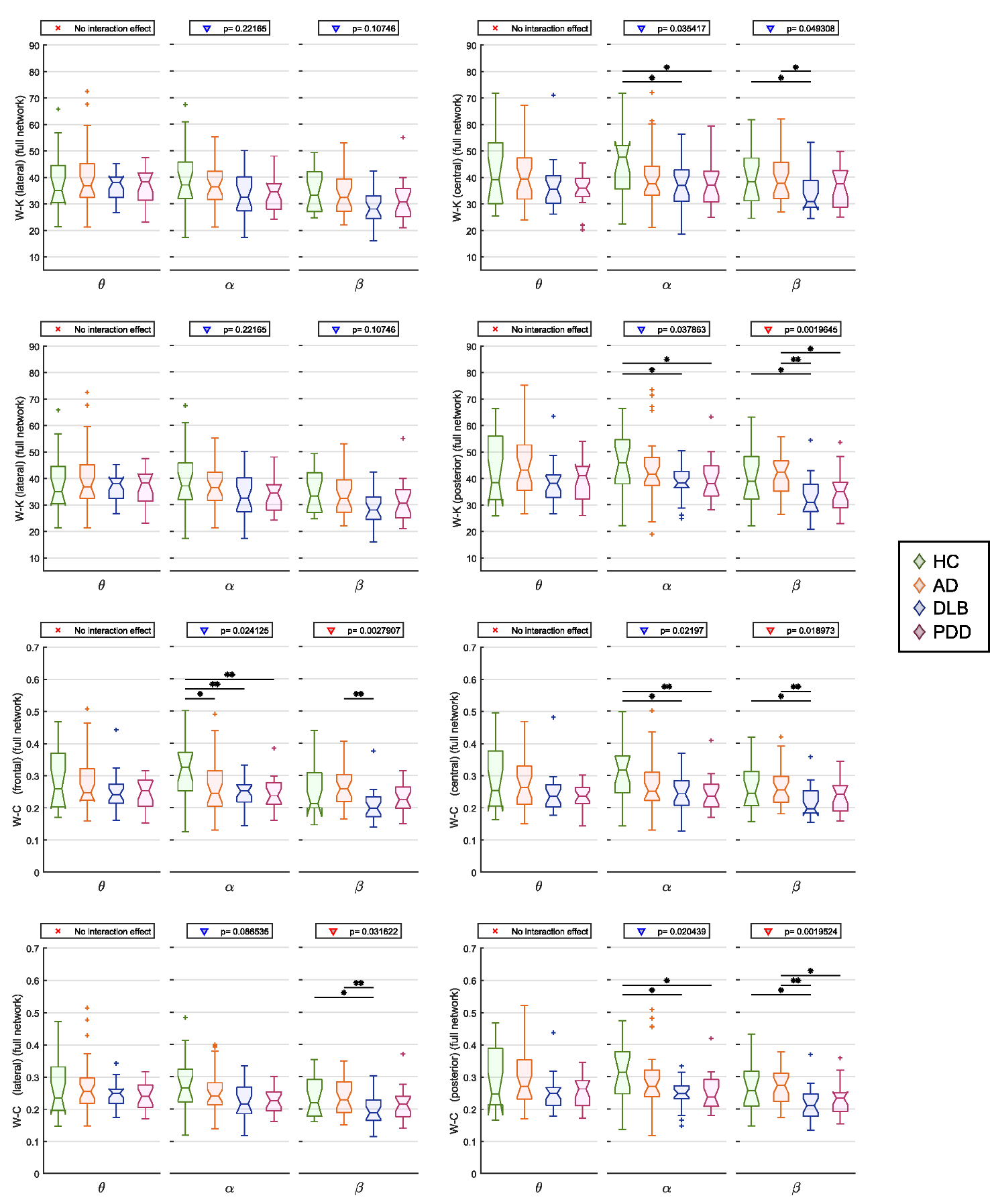


Figure S6. Graph theory analysis from local weight-based network measures over non-thresholded matrices. y-axis: local network measure; x-axis: frequency band of interest (θ: 4-7.5 Hz, α: 8-13.5 Hz, β: 14-20.5 Hz). If any interaction was found in the repeated measures ANOVA (within subjects: areas; between subjects: group), the result of the one-way Kruskal-Wallis test (p<0.05) is indicated on top. Red triangle: Kruskal-Wallis test survives Holm-Bonferroni correction (4 areas); *significant two-tailed Mann-Whitney U test post-hoc test (p<0.05); **post-hoc test survives Holm-Bonferroni correction (6 comparisons). Top (A): Weighted node degree. Bottom (B): Weighted clustering coefficient.

1. Correlation between network measures and clinical scores

Figure S7 shows values and plots for significant correlations between network measures and clinical scores. Correlations were computed with nonparametric Spearman’s rank test (p<0.05, uncorrected).

Figure S7 – Significant correlations between weight-based network measures and clinical scores assessed by Spearman test (p<0.05, uncorrected). For each correlation, r^2^ and p-value are shown.

1. Robustness of the network

We tested whether the hubness of the network, i.e. the existence of nodes with high degree, was affected by the pathological condition. For this, we used the weighted connectivity matrices and applied an iterative targeted node attack to the network as described in previous studies (Barabasi and Albert, 1999; Kaiser, et al., 2007; Stam, et al., 2009). For the node attack analysis, we progressively removed nodes with the highest degree one by one and computed the average weight-based characteristic path length (W-L) of the resulting network. Finally, we plotted the node removal percentage vs. W-L graphs. In previous studies, the L values showed an increasing trend with respect to the percentage of nodes removed, reaching a peak and decreasing to zero as more nodes are progressively removed. It has been shown that in scale-free networks, the peak occurs earlier when compared with small-world or random networks (Kaiser, et al., 2007). Scale-free networks compared with other type of networks, are less robust to targeted external attacks, due to their higher hubness. Assuming that healthy brain networks have scale-free properties (Eguiluz, et al., 2005), we expected that the brains from healthy participants show a scale-free behaviour, i.e. the W-L curve shows an earlier peak for the HC groups compared with dementia groups.

Figure S8 – Results of the targeted node attack (edge density=15%). The y axis shows the weight-based characteristic path length, the x axis is the percentage of nodes removed from the network.

Figure S8 shows the outcome of the network node attack analysis at a network density of 15%. As expected, the robustness of network integration, as measured through characteristic path length (L) was associated with disease condition. Although no differences between groups for the fraction of removed nodes, at which the peak in the sequential node elimination curve occurred, were found in the θ and β band. A slight shift towards higher percentages was found in LBDs groups in the α band compared with HCs. Results for network densities at 10% and 20% are reported in Figure S9.

Figure S9 - Results from the targeted node attack (top row: density 10%, bottom row: density 20%). The y-axis shows the weight-based characteristic path length, the x-axis is the percentage of nodes removed from the network.

Whether the human brain has a scale free organisation is still controversial (Eguiluz, et al., 2005; Kaiser, et al., 2007). Due to their higher hubness properties, scale-free networks show a peak in the L at an earlier percentage of removed nodes after external targeted attack (Barabasi and Albert, 1999). In our attack analysis, this peak is delayed at the α band for LBDs compared with HC, which means that in LBDs the hubness of the brain network was negatively affected by the pathology. No difference could be observed at the θ and β band, although differences between groups slightly emerged. Further analysis involving different approaches will be required to effectively assess whether the dementia condition may affect the hubness of the brain network.

1. Diagnostic accuracy

Figure S10 shows the results of the random forest classifier using 5 and 7 folds at cross-validation for the scenarios reported in the main manuscript, i.e. DLB vs AD and LBDs vs HC. Similar classification accuracy was found.

Figure S10 - Receiver operating characteristic (ROC) curves obtained with random forest classifier and computed for the DLB-vs-AD and dementia-vs-HC scenarios. All weighted network measures were used to train the classifier. Computations were performed using cross-validation with four and seven folds (first and second row respectively) and 10 repetitions; for this we used the Scikit-Learn framework (version 0.20.1) and the Imbalanced-Learn (version 0.4.3) library in Python.

We also tested scenarios which do not resulted significant for exploratory purposes, i.e. all dementia groups together vs HC group (Figure S11) and DLB vs PDD (Figure S12). Classification between all dementia groups together vs HC group resulted less accurate than LBDs vs HC reported in the main manuscript (Figure 8). This is due to the fact that only WPLI measured in the α range resulted significantly affected in AD compared with HC.


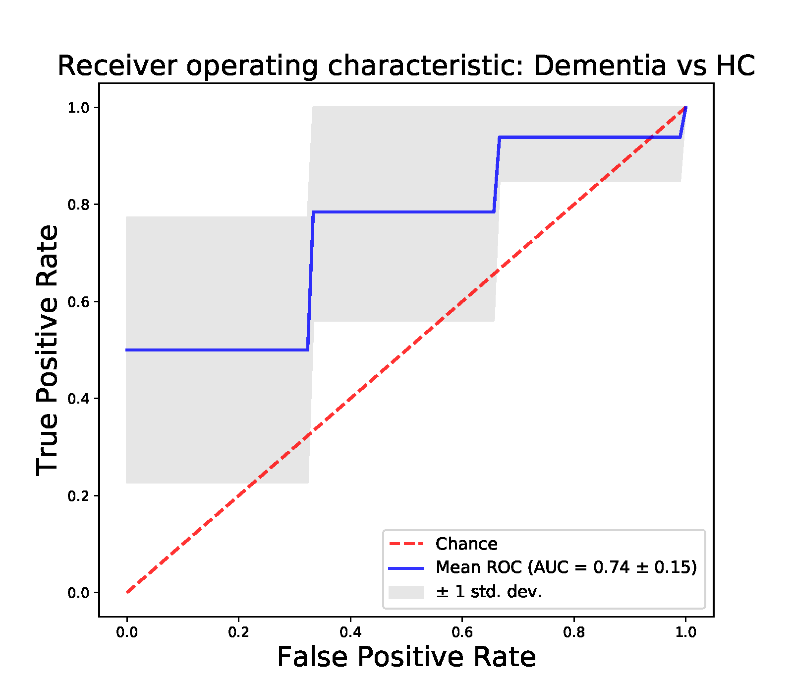


Figure S11 - Receiver operating characteristic (ROC) curves obtained by the random forest classifier and computed for the dementia-vs-HC scenario. All weighted network measures were used to train the classifier. Computations were performed using cross-validation with six folds and 10 repetitions; for this we used the Scikit-Learn framework (version 0.20.1) and the Imbalanced-Learn (version 0.4.3) library in Python.

The overlapping pathology between PDD and DLB affects the classification between the two groups, which resulted driven by chance (Figure S12).


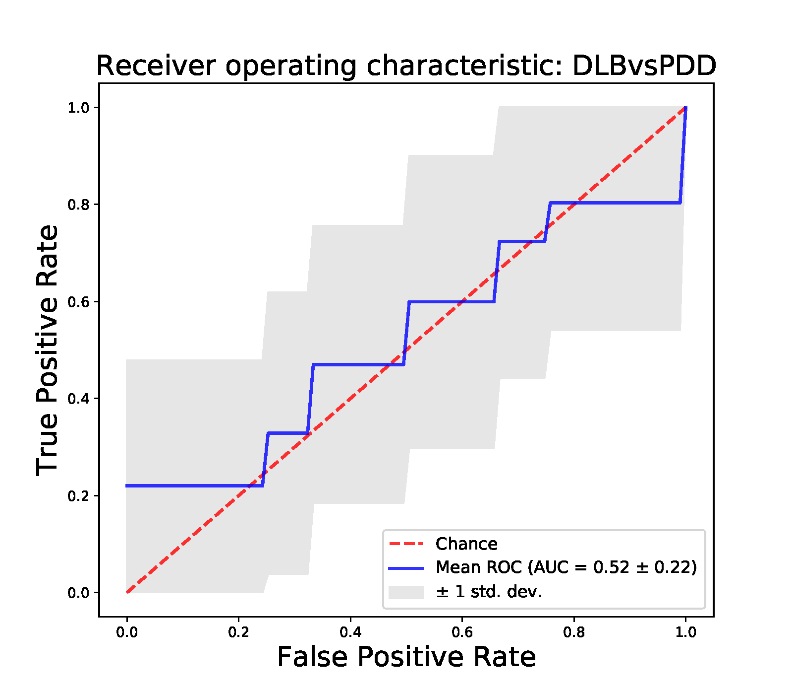


Figure S12 - Receiver operating characteristic (ROC) curves obtained by the random forest classifier and computed for DLB vs PDD scenario. All (weighted) network measures were used to train the classifier. Computations were performed using cross-validation with six folds and 10 repetitions; for this we used the Scikit-Learn framework (version 0.20.1) and the Imbalanced-Learn (version 0.4.3) library in Python.

1. Connectivity and network measures: 10-20 system

We additionally tested whether the same results would be obtained by preserving only the EEG electrodes defined in the standard 10-20 system. This was of our interest as the standard system is used within the clinical framework. As a result, the choice of the EEG cap density resulted not to be crucial for measuring differences between groups in connectivity strength, although some information regarding differences in other network measures is partially lost due to low density setup (Figure S13). This result strengthens the suitability of EEG as possible biomarker, because similarly to our main study, the most important variable for diagnostic discrimination is again the WPLI, as revealed by the random forest classifier (Figure 6).


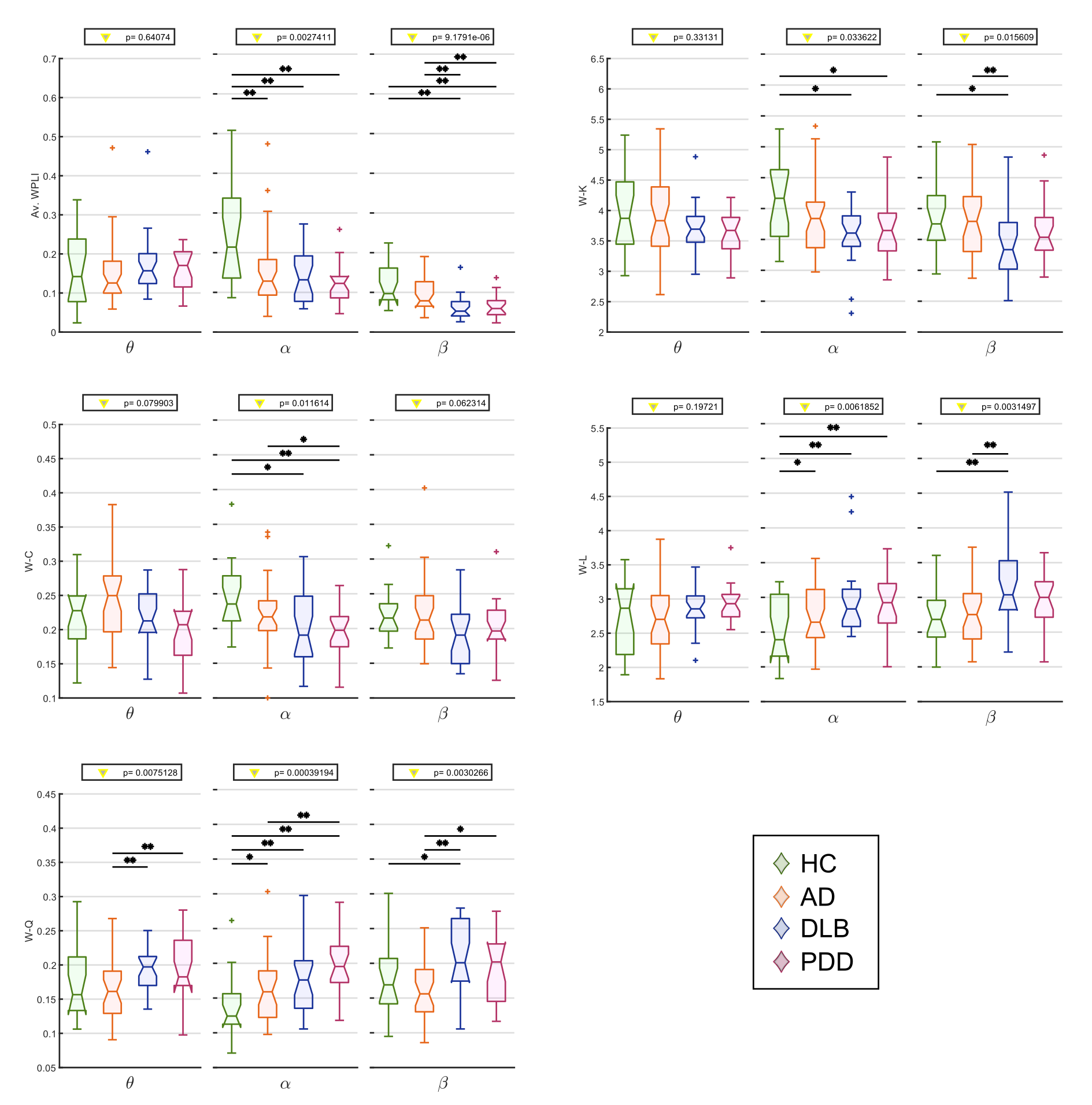


Figure S13. Connectivity and network measures obtained with EEG 10-20 system. y-axis: measure; x-axis: frequency band of interest (θ: 4-7.5 Hz, α: 8-13.5 Hz, β: 14-20.5 Hz). The result of the one-way Kruskal-Wallis test (p<0.05) is indicated on top. *significant two-tailed Mann-Whitney U test post-hoc test (p<0.05); **post-hoc test survives Holm-Bonferroni correction (6 comparisons). Small-worldness is not reported here, as it can be obtained from C and L.

**References**

Barabasi, A.L., Albert, R. (1999) Emergence of scaling in random networks. Science (New York, N.Y.), 286:509-12.

Dijkstra, E.W. (1959) A note on two problems in connexion with graphs. Numerische mathematik, 1:269-271.

Eguiluz, V.M., Chialvo, D.R., Cecchi, G.A., Baliki, M., Apkarian, A.V. (2005) Scale-free brain functional networks. Physical review letters, 94:018102.

Humphries, M.D., Gurney, K. (2008) Network ‘Small-World-Ness’: A Quantitative Method for Determining Canonical Network Equivalence. PloS one, 3:e0002051.

Kaiser, M. (2011) A tutorial in connectome analysis: Topological and spatial features of brain networks. NeuroImage, 57:892-907.

Kaiser, M., Martin, R., Andras, P., Young, M.P. (2007) Simulation of robustness against lesions of cortical networks. European Journal of Neuroscience, 25:3185-3192.

Newman, M.E. (2001) Scientific collaboration networks. II. Shortest paths, weighted networks, and centrality. Physical review E, 64:016132.

Newman, M.E.J., Girvan, M. (2004) Finding and evaluating community structure in networks. Physical Review E, 69:026113.

Onnela, J.-P., Saramäki, J., Kertész, J., Kaski, K. (2005) Intensity and coherence of motifs in weighted complex networks. Physical Review E, 71:065103.

Opsahl, T., Agneessens, F., Skvoretz, J. (2010) Node centrality in weighted networks: Generalizing degree and shortest paths. Social Networks, 32:245-251.

Rubinov, M., Sporns, O. (2010) Complex network measures of brain connectivity: Uses and interpretations. NeuroImage, 52:1059-1069.

Sanz-Arigita, E.J., Schoonheim, M.M., Damoiseaux, J.S., Rombouts, S.A., Maris, E., Barkhof, F., Scheltens, P., Stam, C.J. (2010) Loss of ‘small-world’networks in Alzheimer's disease: graph analysis of FMRI resting-state functional connectivity. PloS one, 5:e13788.

Stam, C.J., de Haan, W., Daffertshofer, A., Jones, B.F., Manshanden, I., van Cappellen van Walsum, A.M., Montez, T., Verbunt, J.P.A., de Munck, J.C., van Dijk, B.W., Berendse, H.W., Scheltens, P. (2009) Graph theoretical analysis of magnetoencephalographic functional connectivity in Alzheimer's disease. Brain : a journal of neurology, 132:213-224.

Watts, D.J., Strogatz, S.H. (1998) Collective dynamics of ‘small-world’networks. Nature, 393:440.
